# Comparison of inpatient vs. outpatient anterior cervical discectomy and fusion: a retrospective case series

**DOI:** 10.1186/1471-2482-9-3

**Published:** 2009-03-05

**Authors:** Jeffrey T Liu, Rudy P Briner, Jonathan A Friedman

**Affiliations:** 1 Departments of Surgery, Neuroscience and Experimental Therapeutics, Texas A&M Health Science Center College of Medicine, College Station, Texas, USA; 2 The Texas Brain and Spine Institute, Bryan-College Station, Texas, USA

## Abstract

**Background:**

Spinal surgery is increasingly being done in the outpatient setting. We reviewed our experience with inpatient and outpatient single-level anterior cervical discectomy and fusion with plating (ACDF+P).

**Methods:**

All patients undergoing single-level anterior cervical discectomy and fusion with plating between August 2005 and May 2007 by two surgeons (RPB or JAF) were retrospectively reviewed. All patients underwent anterior cervical microdiscectomy, arthrodesis using structural allograft, and titanium plating. A planned change from doing ACDF+P on an inpatient basis to doing ACDF+P on an outpatient basis was instituted at the midpoint of the study. There were no other changes in technique, patient selection, instrumentation, facility, or other factors. All procedures were done in full-service hospitals accommodating outpatient and inpatient care.

**Results:**

64 patients underwent ACDF+P as inpatients, while 45 underwent ACDF+P as outpatients. When outpatient surgery was planned, 17 patients were treated as inpatients due to medical comorbidities (14), older age (1), and patient preference (2). At a mean follow-up of 62.4 days, 90 patients had an excellent outcome, 19 patients had a good outcome, and no patients had a fair or poor outcome. There was no significant difference in outcome between inpatients and outpatients. There were 4 complications, all occurring in inpatients: a hematoma one week post-operatively requiring drainage, a cerebrospinal fluid leak treated with lumbar drainage, syncope of unknown etiology, and moderate dysphagia.

**Conclusion:**

In this series, outpatient ACDF+P was safe and was not associated with a significant difference in outcome compared with inpatient ACDF+P.

## Background

Spinal surgery is increasingly being done in the outpatient setting. Reasons suggested for this include the refinement of facilities and systems for ambulatory surgery, increasing utilization of minimally-invasive approaches, increasing utilization of allograft instead of autograft for arthrodesis with associated decrease in graft site pain and morbidity, and improvements in tools and techniques for spinal instrumentation [1-4].

Because of short operative time and moderate postoperative pain, anterior cervical discectomy and fusion with plating (ACDF+P) may be well-suited to be performed in the outpatient setting. However, some potential complications of ACDF+P, including postoperative hematoma, may preclude safely performing the procedure in outpatients. We reviewed our experience with inpatient and outpatient single-level anterior cervical discectomy and fusion with plating (ACDF+P).

## Methods

All patients undergoing single-level anterior cervical discectomy and fusion with plating between August 2005 and May 2007 by one of two surgeons (RPB or JAF) were retrospectively reviewed. All patients underwent anterior cervical microdiscectomy, arthrodesis using structural allograft, and titanium plating. The technique used is a modification of the procedure as originally described by Smith and Robinson [5]. Briefly, a transverse right sided cervical incision was used for exposure. After incision of the intervertebral disc and removal of anterior osteophytes, distraction pins placed in the vertebral bodies were used for distraction. Using the operating microscope, all disc material and posterior osteophytes were removed. The posterior longitudinal ligament was excised routinely at the intervertebral space. The bony endplates were prepared with rasps and curettes. Machine-fabricated cadaveric cortical allograft was then tapped securely into the intervertebral disc space. All patients had anterior titanium plating with two screws at each vertebral body. The most common plates used were made by Stryker (Reflex hybrid plate, Stryker, Kalamazoo MI) and Depuy (Slim Loc and Skyline plates, Depuy Spine, Raynham MA).

One-hundred and nine consecutive patients underwent surgery between August 2005 and May 2007, and were analyzed in this study. An intentional change from doing ACDF+P on an inpatient basis to doing ACDF+P on an outpatient basis was instituted in July 2006, roughly corresponding to the midpoint of the study. There were no other known changes in technique, patient selection, instrumentation, facility, or other factors. All procedures were done in full-service hospitals accommodating outpatient and inpatient care. Postoperative radiographs were planned in both inpatients and outpatients with at two and eight weeks postoperatively. Based on retrospective chart review, complications were recorded and outcome was assessed at longest follow-up (mean 62.4 days postoperatively, range 7–208 days). An excellent outcome was defined as a complete resolution of symptoms. A good outcome was defined as a partial resolution of symptoms with non-debilitating residual symptoms. A fair outcome was defined as no improvement in symptoms. A poor outcome was defined as an exacerbation of symptoms. Statistical analysis was done using a two-sided test for equality of proportions. The study was reviewed and approved by the St. Joseph Hospital Institutional Review Board (Bryan, TX).

## Results

Sixty-four patients (58.7%) underwent ACDF+P as inpatients, while 45 patients (41.3%) underwent ACDF+P as outpatients. During the time period when outpatient surgery was performed routinely, 17 patients (27.4%) were treated as inpatients due to medical comorbidities (14), older age (1), and patient preference (2). No patient in whom outpatient surgery was planned was unexpectedly converted to inpatient. At a mean follow-up of 62.4 days, 90 patients had an excellent outcome (82.6%), 19 patients had a good outcome (17.4%), and no patients had a fair or poor outcome. There was no significant difference in outcome between inpatients and outpatients (see additional file 1: Table 2; p = 0.14). Preoperative presentation in patients treated as inpatients included myelopathy in 42%, radiculopathy in 41%, a combination of myelopathy and radiculopathy in 14%, and neck pain alone in 3%. Among patients treated as outpatients, 60% presented with radiculopathy, 24% with myelopathy, 16% with a combination of symptoms, and none with neck pain alone. Ten patients had had previous cervical spine surgery, all at different levels than the included operation (8 inpatients, 2 outpatients).

The average age of the inpatient group was 56.06 years, while the average age of the outpatient group was 48.73 years. There were 120 total major medical comorbidities in the 64 inpatients (1.875 average major medical comorbidities per inpatient), compared to 63 total major medical comorbidities in 45 outpatients (1.4 average major medical comorbidities per outpatient). Comparison of the types of major medical comorbidities between the two groups is shown in Table 1 (see additional file 1: Table 1). The most common medical comorbidites in both groups were hypertension and hypercholesterolemia. The higher average age and number of comorbidities in the inpatient group reflects the greater likelihood of selection for inpatient surgery due to increased number or severity of medical comorbidities, or in one patient selection for inpatient surgery based on advanced age alone. Fourteen of the 17 patients that underwent inpatient ACDF+P during the time period when routine outpatient surgery was planned did so due to their medical comorbidities.

There were 4 complications total (3.7%), all in the inpatient group. One of the patients who had a complication was one of the seventeen patients treated as an inpatient due to medical comorbidities, during the period when outpatient surgery was planned; this patient had moderate dysphagia. These symptoms had completely resolved at follow-up on post-operative day 57. One patient had intermittent syncope which developed 38 days postoperatively. The etiology was not determined, and the symptoms had resolved on follow up at postoperative day 59. One patient had a symptomatic cerebrospinal fluid leakage, due to surgical excision of the dura with a large calcified central disc herniation. The patient was treated with lumbar drainage for 6 days, without recurrence of CSF leakage. One patient developed a wound hematoma at one week postoperatively. The patient presented with neck swelling and dysphagia, but had no airway compromise or respiratory distress. This required hospitalization and hematoma evacuation, performed without complication. This patient had no preoperative bleeding diathesis, but had a more extensive exposure to remove preexisting hardware from C6-T1, prior to performing ACDF+P and C5-C6. No patients died or suffered permanent procedure-related morbidity during the follow-up period.

## Discussion

The trend towards ambulatory surgery for procedures previously limited to the inpatient setting is well established [6-11]. Trends in spinal surgery towards less invasive approaches and exposures, as well as modified anesthetic and pain management techniques have resulted in increasing numbers of spinal surgeries being performed in the outpatient setting [12-16]. Anterior cervical discectomy and fusion with plating (ACDF+P) is one of the most common spinal and neurosurgical procedures performed [17-19]. Traditionally, ACDF+P has been performed in the inpatient setting, and this was the practice at our institution. Based on our experience with rapid patient recovery from anesthesia, satisfactory pain control, short operative times, and rare complications in the initial 24 hours, we undertook a systematic and mutually agreed change to routinely perform all ACDF+P as outpatient procedures, unless significant medical risk factors existed. Because we did not intentionally change any other known treatment or selection related factors, the paradigm change provided an opportunity to compare inpatient with outpatient surgery paradigms for ACDF+P in reasonably comparable patient groups.

The complication rate for both groups was low, as there were 4 complications total, all in the inpatient group. Because inpatients had higher baseline risk factors including older age and more medical comorbidities, the increased number of complications in the inpatient group is not surprising and does not seem likely to be reflective of an inherent increased risk in inpatient compared to outpatient surgery. Our rate of postoperative dysphagia was quite low in comparison to previous studies, which could reflect limited sensitivity of the retrospective study design to detect mild dysphagia. A central issue in consideration of inpatient vs. outpatient surgery for patients undergoing ACDF+P relates to the risk of postoperative hematoma, and the theoretical risk that diagnosis and definitive treatment of this complication could be delayed in the outpatient setting [20-24]. Because of potentially fatal airway compromise related to postoperative cervical hematoma, this issue merits serious consideration despite the low incidence of this complication

In the literature, the reported risk of hematoma after ACDF ranges from 0.2% to 7.9% [17,25-27]. In our series, one patient (0.9%) developed a postoperative hematoma. Importantly, the hematoma developed one week postoperatively, and thus evaluation and treatment were not affected by choice of inpatient vs. outpatient initial management. Our data does not support routine inpatient surgery for ACDF+P to watch for development of postoperative hematoma. However, given the low incidence of this complication, the possibility that a much larger sample size of patients could demonstrate that the choice of inpatient vs. outpatient surgery for ACDF+P could adversely influence the evaluation and treatment of postoperative hematoma cannot be excluded.

Existing literature supports safety and efficacy of anterior cervical discectomy in the outpatient setting. Silvers et al. and Erickson et al. found no compromise in safety and efficacy of anterior cervical discectomy and fusion when performed as an outpatient [24,28]. However, anterior cervical plating was not used in these series. Stieber et al. found fewer complications in outpatients undergoing ACDF+P, in a highly selected low-risk cohort of patients [29]. Villavicencio et al. evaluated outpatient and 23-hour admission ACDF+P and compared to a historical cohort of patients treated as inpatients, finding no difference in complication rates [30]. The findings of our study are consistent with the reported literature, demonstrating no increase in complication rate or worse outcomes in patients treated as outpatients. Our study has unique value in comparison to other published studies because it involves direct comparison of inpatient and outpatient ACDF+P without significant variation in other treatment related variables. In our study patients with myelopathy were also considered and successfully treated as outpatients – 24% of outpatients treated in our series presented with myelopathy, while 16% presented with a combination of myelopathy and radiculopathy.

While not a prospective randomized trial, the absence of any other significant systematic changes in surgical technique or other features of management over the time period of the study allows meaningful comparison of the two groups. Selection bias certainly exists, since 17 patients during the time period of routine outpatient surgery were treated as inpatients due to medical comorbidities and older age. Thus, our experience can only be generalized when similar selection of high risk patients for inpatient care is also utilized. This selection bias almost certainly accounts for the lower complication rate in the outpatient group compared with the inpatient group. Even in the setting of a randomized trial, patients with significant medical comorbidities would likely be excluded from eligibility for randomization.

Because it is unlikely that a randomized trial would be done for reasons of cost and efficiency, a comparison such as ours, while not randomized, may be the most representative data realistically achievable. In our study, the selection of certain patients to be treated as inpatients during the planned outpatient period of the study because of comorbidities causes dissimilarity between the comparison groups. However, it is likely impossible or unethical to enroll patients in a study when the treating physician believes that the study parameters increase risk to the patient. Even if the inpatients treated during the planned outpatient period are excluded, the results of the study are unchanged. The optimal age cutoff or number and severity of comorbidities to determine whether inpatient or outpatient paradigms should be employed cannot be determined from these data.

Anterior cervical discectomy with plating performed in the outpatient setting may carry significant total cost savings, compared with inpatient treatment. Chi et al. estimates the cost of single or double-level ACDF to be from $10,000 to $15,000, which includes materials, surgeon labor fees, and hospital stay [31]. Cost savings from $4,000 to $8,000 with outpatient ACDF versus inpatient ACDF have been reported by Erickson et al [27]. With 150,000 ACDF procedures performed annually in the United States [32], total health cost savings associated with converting inpatient to outpatient procedures could exceed $100 million annually.

## Conclusion

In this series, outpatient ACDF+P was not associated with a significant difference in outcome or complications compared with inpatient ACDF+P. Optimal criteria regarding age and medical comorbidities for selecting patients for inpatient vs. outpatient ACDF+P remain unknown.

## Competing interests

The authors declare that they have no competing interests.

## Authors' contributions

JTL conducted the research and drafted the manuscript. RPB performed the surgeries. JAF performed the surgeries, conceived the idea of the study, and participated in its design and coordination. All authors read and approved the final manuscript.

## Pre-publication history

The pre-publication history for this paper can be accessed here:



## Supplementary Material

Additional file 1 Tables. Table 1 – Demographic data. Table 2 – Outcomes and complications Click here for file
