# Supplementary material for: Comparison of inpatient vs. outpatient anterior cervical discectomy and fusion: a retrospective case series
Source: BMC Surg. 2009 Mar 5;9:3. doi: 10.1186/1471-2482-9-3 (PMC2657115; doi:10.1186/1471-2482-9-3)
Supplement: Additional file 1 — Tables. Table 1 – Demographic data. Table 2 – Outcomes and complications [file 1471-2482-9-3-S1.doc]

# **Table 1**

# *Demographic data*

_____________________________________________________________________________________________________________

|  | n | Average age | Females | Preoperative symptoms | Vertebral level | Medical comorbidities |
| --- | --- | --- | --- | --- | --- | --- |
| Inpatients | 64 (59%) | 56.06 (22 – 89) | 27 (42%) | Myelopathy – 27 (42%)Radiculopathy – 26 (41%)Combination – 9 (14%)Neck pain – 2 (3%) | C5-6 – 20 (31%)C6-7 – 20 (31%)C4-5 – 14 (22%)C3-4 – 8 (13%)C7-T1 – 2 (3%) | Hypertension – 28 (44%)High cholesterol – 20 (31%)Arthritis – 18 (28%)Diabetes – 13 (20%)Depression – 9 (14%)Migraines – 9 (14%)Heartburn/reflux – 7 (11%)Obesity – 5 (8%)Cancer – 4 (6%)Stroke – 3 (5%)Hepatitis – 2 (3%)Osteoporosis – 2 (3%) |
| Outpatients | 45 (41%) | 48.73 (23 – 64) | 14 (31%) | Radiculopathy – 27 (60%)Myelopathy – 11 (24%)Combination – 7 (16%)Neck pain – 0 (0%) | C6-7 – 20 (44%)C5-6 – 16 (35%)C4-5 – 5 (11%)C3-4 – 3 (7%)C7-T1 – 1 (2%) | Hypertension – 15 (33%)Depression – 9 (20%)Heartburn/reflux – 9 (20%)High cholesterol - 9 (20%)Migraines – 6 (13%)Arthritis – 3 (7%)Hepatitis - 3 (7%)Osteoporosis – 3 (7%)Diabetes – 2 (4%)Obesity – 2 (4%)Cancer – 1 (2%)Stroke - 1 (2%) |

n = number of patients; ranges are presented in parenthesis

**Table 2**

*Outcomes and complications*

*_____________________________________________________________________________________________________________*

|  | Excellent outcome (complete resolution of symptoms) | Good outcome (partial resolution of symptoms with some residual symptoms) | Fair outcome (no improvement in symptoms) | Poor (exacerbation of symptoms) | Complications |
| --- | --- | --- | --- | --- | --- |
| Inpatients – 64 (59%) | 50 (78.1%) | 14 (21.9%) | 0 (0%) | 0 (0%) | 4 (6%) (CSF leak, moderate dysphagia, hematoma, syncope) |
| Outpatients – 45 (41%) | 40 (88.9%) | 5 (11.1%) | 0 (0%) | 0 (0%) | 0 (0%) |
